# Supplementary material for: High-quality mesoporous graphene particles as high-energy and fast-charging anodes for lithium-ion batteries
Source: Nat Commun. 2019 Apr 1;10:1474. doi: 10.1038/s41467-019-09274-y (PMC6443805; doi:10.1038/s41467-019-09274-y)
Supplement: Supplementary file 1 — Supplementary Information [file 41467_2019_9274_MOESM1_ESM.pdf]

Supplementary Information

High-quality mesoporous graphene particles as high-energy and fast-charging anodes for lithium-ion batteries

Mo *et al*.

Supplementary Figures

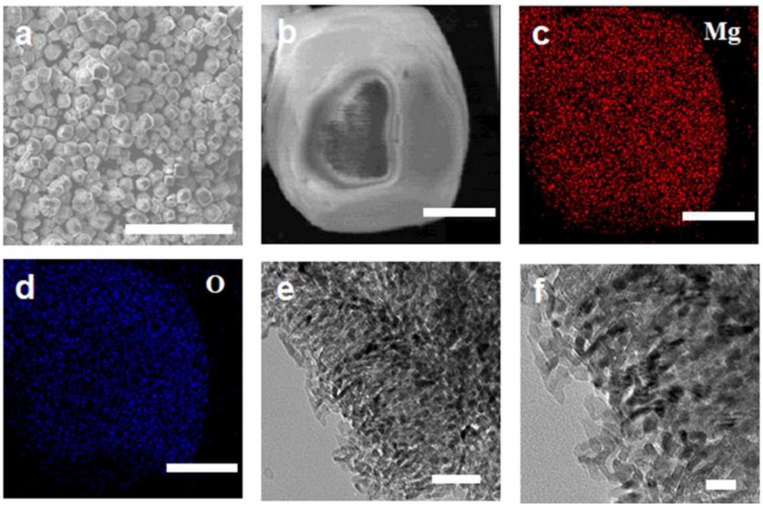

Supplementary Figure 1. Morphology and structure of MgO particles. (a) SEM image of the MgO particles. Scale bars: a 50 µm. **(b–d)** A SEM image and EDS elemental maps of Mg and O of a MgO particle. Scale bars: b 2.5 µm; c 2.5 µm; d 2.5 µm. **(e, f)** TEM images of the MgO particles. Scale bars: e 50 nm; f 20 nm.

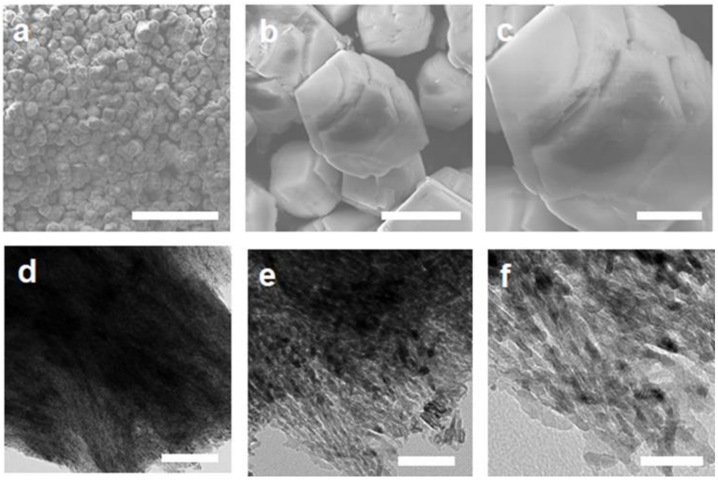

Supplementary Figure 2. Morphology and structure of graphene-coated MgO particles. **(a–c)** SEM and **(d–f)** TEM images of the graphene coated MgO particles. Scale bars: a 50 µm; b 5 µm; c 2 µm; d 200 nm; e 50 nm; f 20 nm.

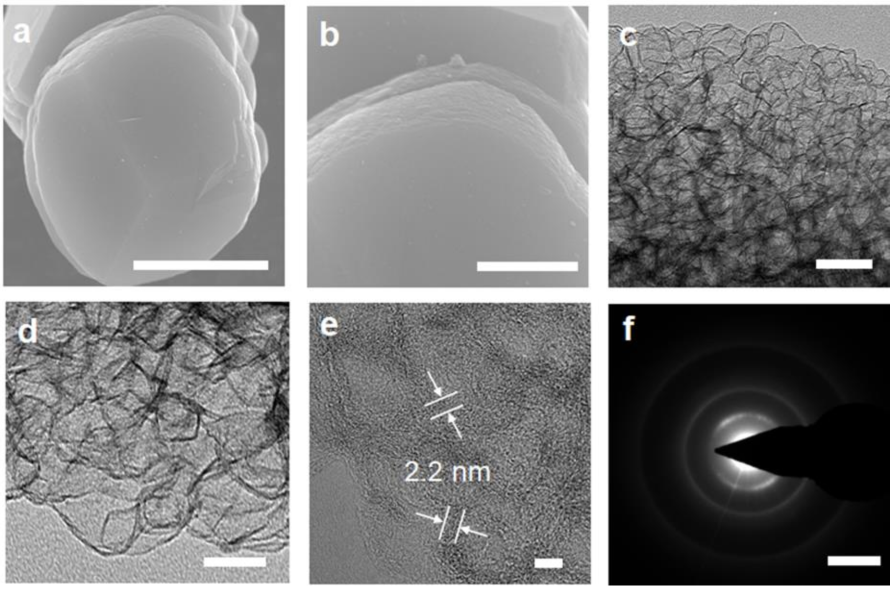

Supplementary Figure 3. Morphology and structure of NMG particles. (a,b) SEM images of the HNMG particles. Scale bars: a 4 μm; b 2 μm. **(c,d)** TEM images of the NMG. Scale bars: c 50 nm; d 20 nm. **(e)** High-resolution TEM of the NMG particles. Scale bars: e 5 nm. **(f)** The electronic diffraction pattern corresponding to the NMG particles. Scale bars: f 5 1/ nm.

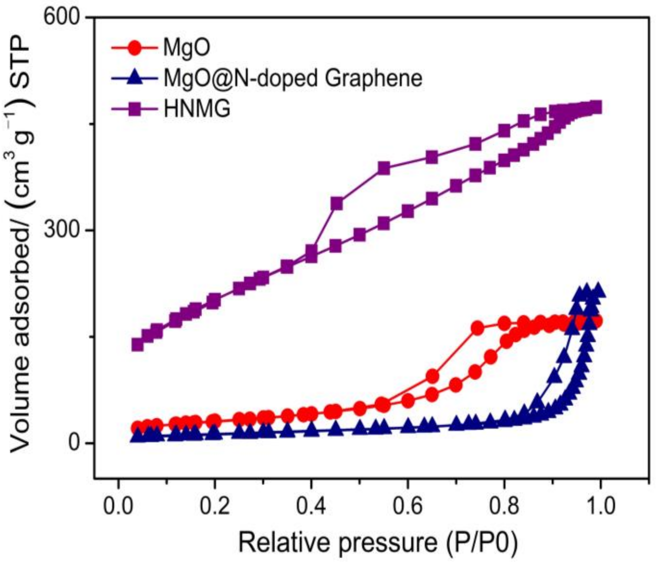

Supplementary Figure 4. N_2 adsorption/desorption isotherms for MgO particles, graphene-deposited MgO particles, and HNMG particles.

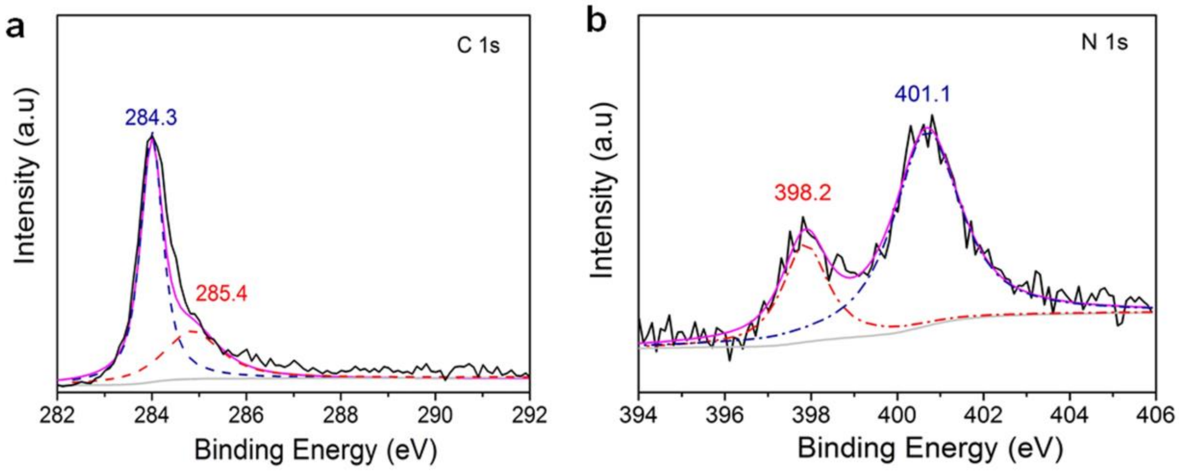

Supplementary Figure 5. Structure of HNMG particles. (a) High resolution C1s XPS spectra. **(b)** High resolution N1s XPS spectra.

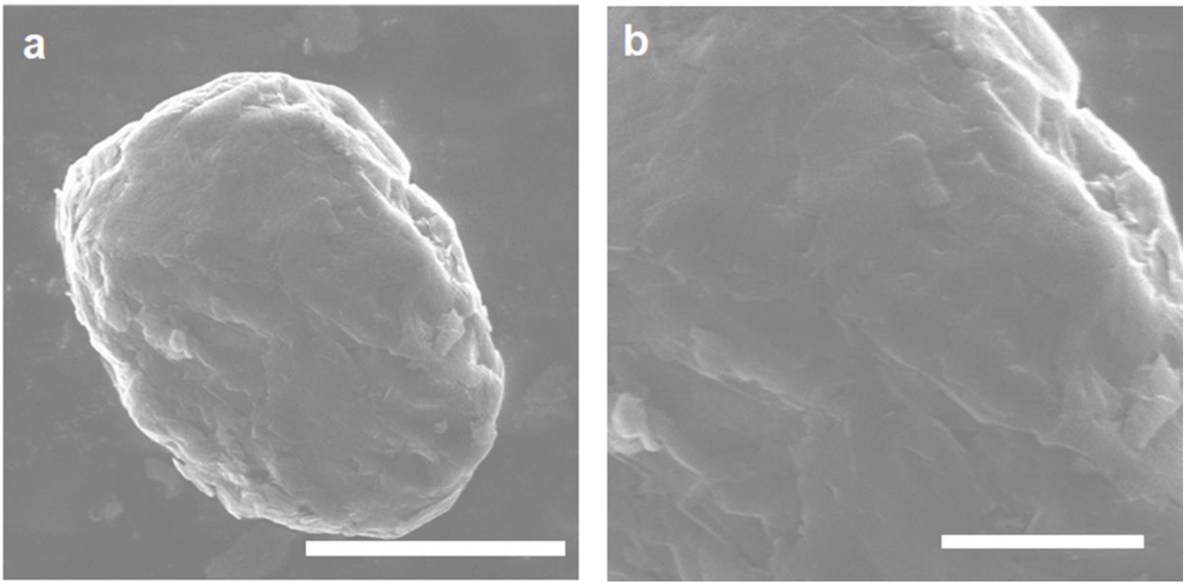

Supplementary Figure 6. Morphology of graphite particles. (a,b) SEM images of the graphite particles. Scale bars: a 5 μm; b 2 μm.

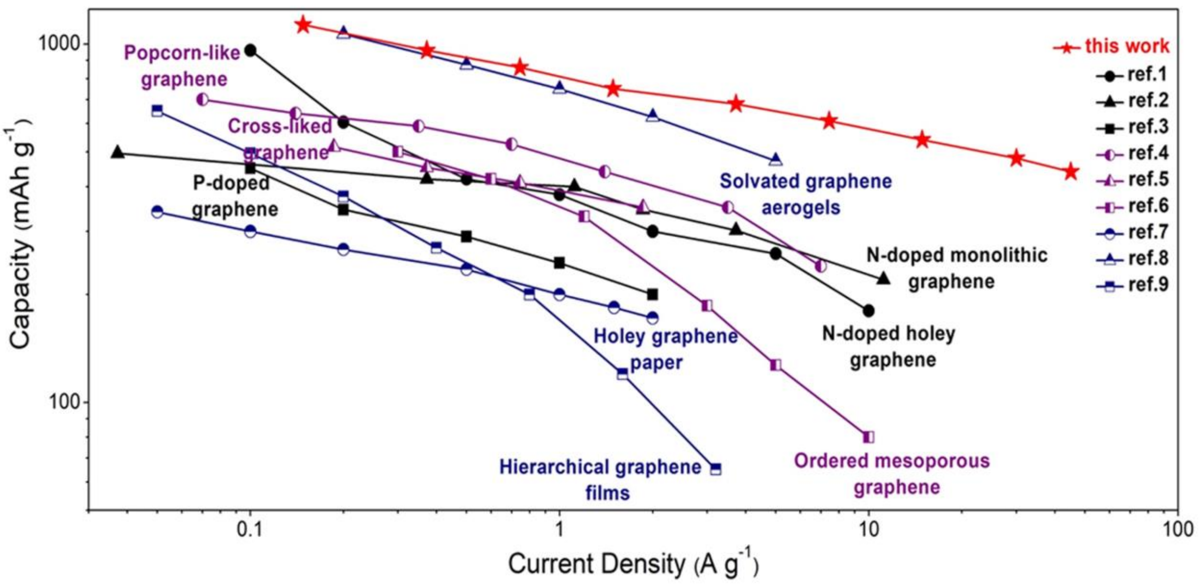

Supplementary Figure 7. The comparison of specific capacity vs current density of the HNMG electrodes with representative graphene anodes reported.

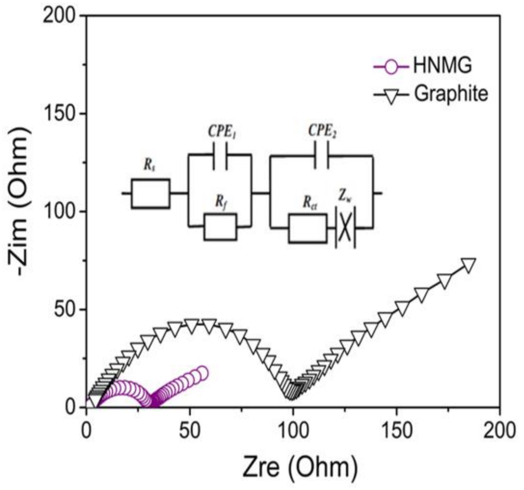

Supplementary Figure 8. Nyquist plots of HNMG and graphite anode obtained by applying a sine wave with amplitude of 5.0 mV over the frequency range from 100 kHz to 0.01 Hz and an equivalent circuit used to fit the Nyquist plot. The circuit elements are composed of the solution resistance (R_s), the charge-transfer resistance (R_{ct}), the contact resistance (R_f) and the Warburg impedance (Z_w), respectively.

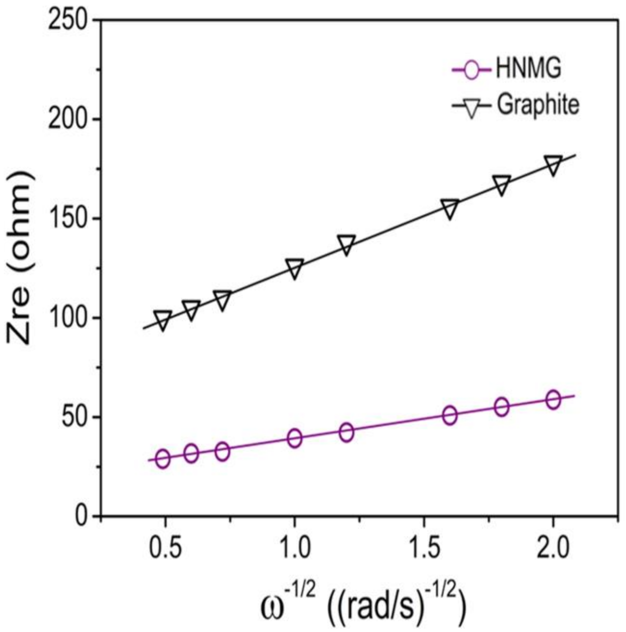

Supplementary Figure 9. The relationship between the real part of the impedance spectra (Z_{re}) vs. $\omega^{-1/2}$ in the low-frequency region, where ω is the angular frequency ($\omega = 2\pi f$).

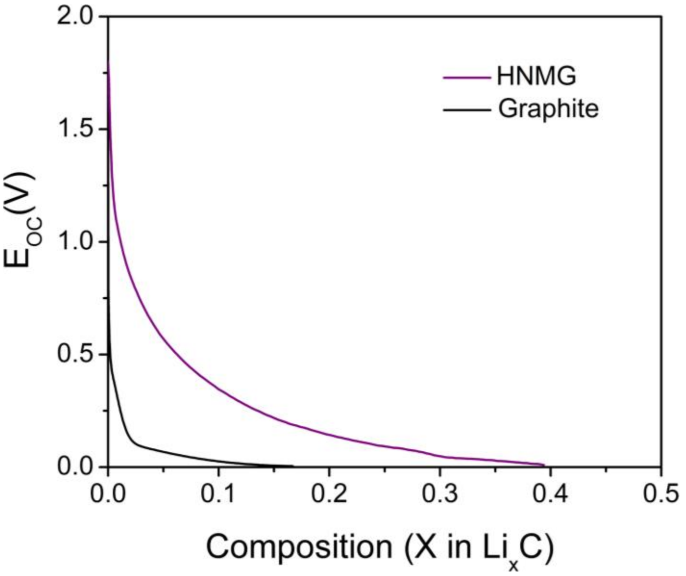

Supplementary Figure 10. Plots of the open-circuit potential (E_{oc}) of a HNMG and a graphite electrode vs. their molar fraction of lithium (x) in the lithiated electrodes (Li_xC).

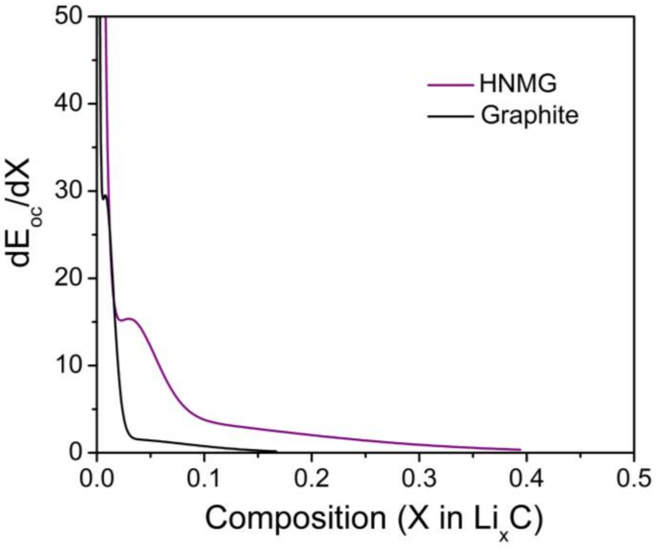

Supplementary Figure 11. The gradient of the coulometric titration curve (dEoc/dx) vs. the lithium composition *x* in the lithiated HNMG and graphite electrodes.

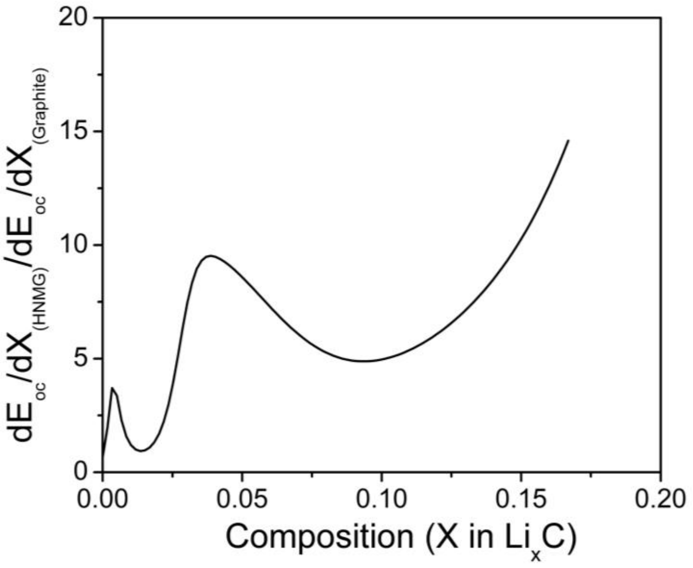

Supplementary Figure 12. A plot of the ratio of $dEoc/dx_{(HNMG)}$ and $dEoc/dx_{(Graphite)}$ vs. the composition *x* in the lithiated HNMG and graphite electrodes.

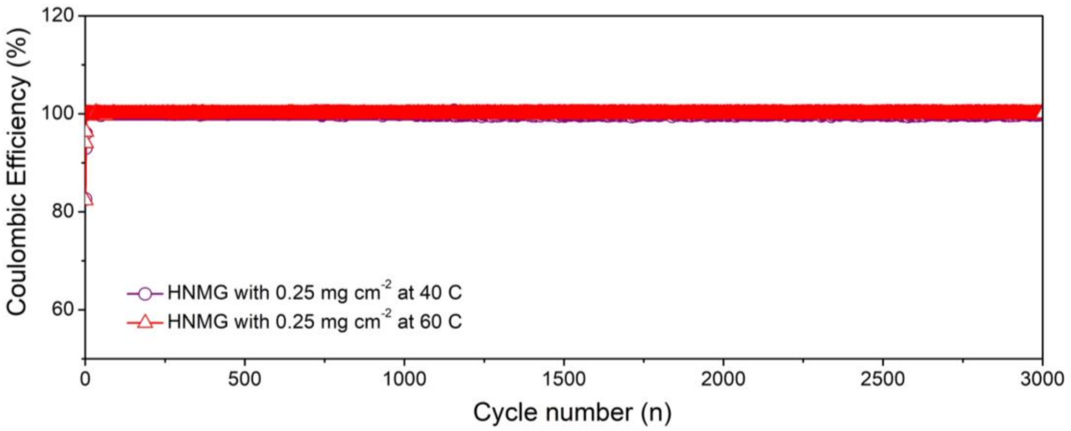

Supplementary Figure 13. Coulombic efficiency for HNMG electrodes with a mass loading of 0.25 mg cm^{-2} at rates of 40 C and 60 C for 3000 cycles.

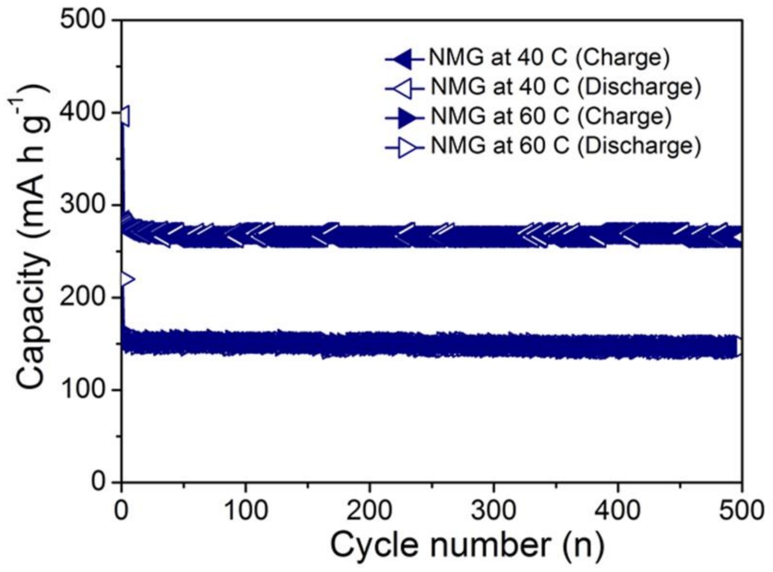

Supplementary Figure 14. Cycling stability of the NMG electrode at rates of 40 and 60 C for 500 cycles.

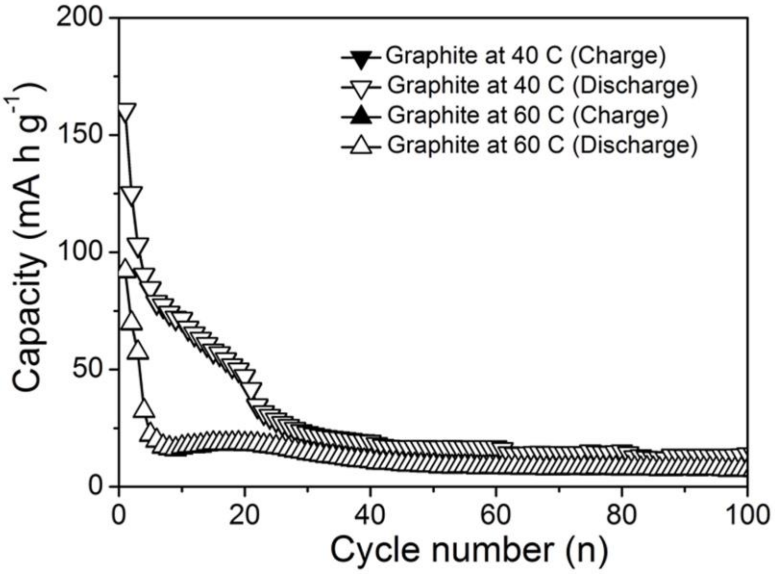

Supplementary Figure 15. Cycling stability of the graphite electrode at rates of 40 and 60 C for 100 cycles.

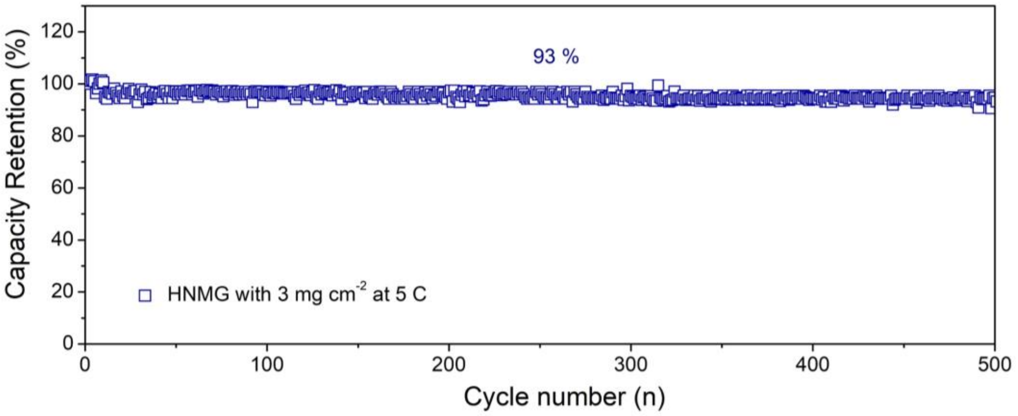

Supplementary Figure 16. Cycling performance for the HNMG electrode at a rate of 5 C for 500 cycles. The mass loading is 3 mg cm^{-2}.

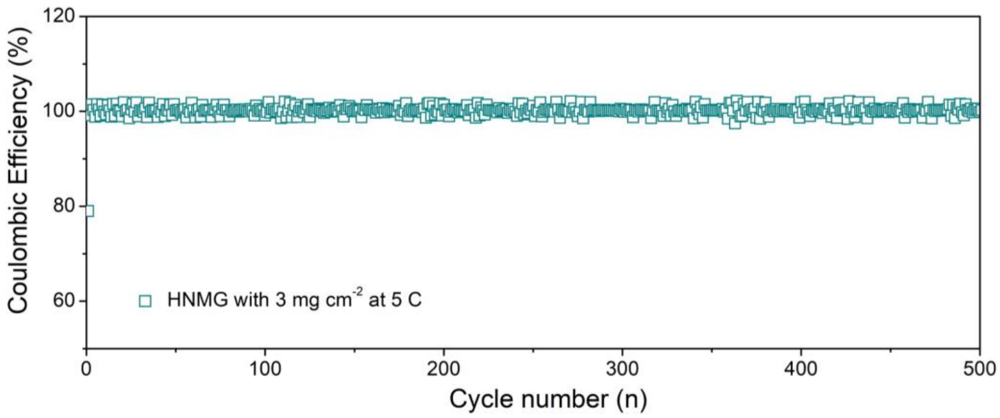

Supplementary Figure 17. Coulombic efficiency for the HNMG electrode at a rate of 5 C for 500 cycles. The mass loading is 3 mg cm^{-2}.

Supplementary Tables

Supplementary Table 1. Tap density of various materials.

Materials	Tap density (g cm^{-3})	Packing density (g cm^{-3})
HNMG	0.63	0.83
NMG	0.52	0.72
Graphite	1.0	1.5

Supplementary Table 2. Electrochemical performance of reported typical graphene-based anode materials in comparison with our results.

Materials	Carbon black (%)	Mass loading (mg cm^{-2})	Current density (A g^{-1})	Capacity (mAh g^{-1})	Current density (A g^{-1})	Capacity (mAh g^{-1})	Reference
Holey Graphene Paper	0	0.3	0.05	403	2	178	[7]
Laser-scribed Graphene Paper	0	NA	0.37	545	1.86	335	[10]
Phosphorus-Doped Graphene	15	NA	0.1	460	2	190	[3]
3D Fe_3O_4/Graphene Foam	0	NA	0.37	785	5.5	400	[11]
3D Fe_3O_4/Graphene	10	NA	0.15	856	4.8	363	[12]
Mesoporous Graphene Sheet	10	NA	0.1	770	5	255	[13]
3D Fe_2O_3/Graphene	0	0.8	0.2	871	5	587	[14]
3D MoS_2/Graphene	10	1.5	0.07	1216	1.86	711	[15]
3D Sn/Graphene	10	NA	0.2	1022	5	459	[16]
3D WS_2/Graphene	0	1.9	0.1	693	1	394	[17]
Solvated Graphene Frameworks	0	2	0.2	1034	5	472	[8]
Nitrogen-Doped Holey Graphene	10	1	0.1	989.5	10	161	[1]
Nitrogen-Doped Graphene	15	NA	0.1	655	5	150	[18]
Nb_2O_5/HGF	10	1	0.2	160	20	75	[19]
Graphene Ball	0	NA	0.07	700	7	240	[4]
**HMNG Particles**	**0**	**1**	**0.15**	**1138**	**45**	**440**	**This work**
		**3**		**1126**	**30**	**361**	
		**6**		**1109**	**30**	**221**	

Supplementary Table 3. Electrochemical performance of reported typical various carbon-based materials prepared by template method in comparison with our results.

Materials	Template	Mass loading (mg cm^{-2})	Current density (A g^{-1})	Capacity (mAh g^{-1})	Current density (A g^{-1})	Capacity (mAh g^{-1})	Reference
Graphene Ball	SiO_2	NA	0.07	700	7	240	[4]
Mesoporous Graphene Sheet	AAO	NA	0.1	770	5	255	[13]
3D Macroporous Carbon Monolith	PMMA	NA	0.015	299	0.15	125	[20]
Hollow Carbon Sphere	SiO_2	NA	0.074	320	5.58	210	[21]
Hierarchically Porous Carbon Monolith	SiO_2	2	0.372	540	22.32	70	[22]
Ordered Multimodal Porous Carbon Sphere	SiO_2	NA	0.1	903	1	758	[23]
Hollow Mesoporous Carbon Sphere	SiO_2	NA	0.074	268	3.72	100	[24]
Nitrogen-Rich Mesoporous Carbon Plate	$CaCO_3$	1	0.1	900	0.8	400	[25]
Nitrogen-Rich Porous Carbon Sphere	SiO_2	NA	0.5	542	3	215	[26]
Porous Carbon Fiber	MgO	NA	0.25	1020	4	355	[27]
**HMNG Particles**	**0**	**1**	**0.15**	**1138**	**45**	**440**	**This work**
		**3**		**1126**	**30**	**361**	
		**6**		**1109**	**30**	**221**	

Supplementary Notes

According to the Warburg impedance model, $Z_{re} = \delta\ \omega^{-1/2}$, where Z_{re} is the real part of the resistance, δ is the Warburg prefactor and ω is the angular frequency.[28] δ is related to the diffusion coefficient of lithium ions (D) by

$$\delta = \frac{V_m \frac{dE_{oc}}{dx}}{FA(2D)^{0.5}}$$

where V_m is the molar volume of the lithiated HNMG or graphite, F is the Faraday constant, A is the electrode area, and dE_{oc}/dx is the gradient of the coulometric titration curve. dE_{oc}/dx can be obtained from a plot of the open-circuit potential (E_{oc}) vs. the molar fraction of lithium "x" in the HNMG or graphite at each charged state.

Assuming that graphite and graphene have a similar molar volume (Vm) and that both the electrode have a similar electrode area (A), and F is the Faraday constant, therefore, δ and dE_{oc}/dx related to the diffusion coefficient of lithium ions (D) by

$$\frac{D(HNMG)}{D(Graphite)} = [\frac{\frac{dE_{oc}}{dx(HNMG)}}{\frac{dE_{oc}}{dx(Graphite)}} \frac{\delta(Graphite)}{\delta(HNMG)}]^2$$

Based on the δ measured (16 $\Omega/s^{0.5}$ and 64 $\Omega/s^{0.5}$ for the HNMG and graphite electrode, respectively), the ratio of δ between the HNMG and the graphite electrode is 1/4. Based on the results shown in Supplementary Figs. 9–12, the ratio of dE_{oc}/dx for the HNMG electrode vs the graphite electrode varies in the range of 5 to 15. Accordingly, the ratio of the diffusion coefficient of lithium ion in the HNMG and graphite electrode, $[\frac{D(HNMG)}{D(Graphite)}]$, can be estimated with a number ranging from 400 to 3600. The diffusion coefficient of lithium ion in the HNMG electrode, based on the calculation above, is 2 to 3 orders of magnitude higher than that of the graphite electrode.

Supplementary References

1. Xu, J. T., Lin, Y., Connell, J. W. & Dai, L. M. Nitrogen-doped holey graphene as an anode for lithium-ion batteries with high volumetric energy density and long cycle life. *Small*. **11**, 6179–6185 (2015).

2. Liu, X. X. et al. A low-cost and one-step synthesis of N-doped monolithic quasi-graphene films with porous carbon frameworks for Li-ion batteries. *Nano Energy*. **17**, 43–51 (2015).

3. Zhang, C. Z., Mahmood, N., Yin, H., Liu, F. & Hou, Y. L. Synthesis of phosphorus-doped graphene and its multifunctional applications for oxygen reduction reaction and lithium ion batteries. *Adv. Mater*. **25**, 4932–4937 (2013).

4. Son, I. H. et al. Graphene balls for lithium rechargeable batteries with fast charging and high volumetric energy densities. *Nat. Commun*. **8**, 1561 (2017).

5. Tang, J. J. et al. Silica-assistant synthesis of three-dimensional graphene architecture and its application as anode material for lithium ion batteries. *Nano Energy*. **8**, 62–70 (2014).

6. Jiao, Y. C. et al. Highly ordered mesoporous few-layer graphene frameworks enabled by Fe_3O_4 nanocrystal superlattices. *Angew. Chem. Int. Ed*. **54**, 5727–5731 (2015).

7. Zhao, X., Harner, C. M., Kung, M. C. & Kung, H. Flexible holey graphene paper electrodes with enhanced rate capability for energy storage applications. *ACS Nano*. **5**, 8739–8749 (2011).

8. Xu, Y. X. et al. Solvated graphene frameworks as high-performance anodes for lithium-ion batteries. *Angew. Chem. Int. Ed*. **54**, 5345–5350 (2015).

9. Ye, M. H. et al. Uniquely arranged graphene-on-graphene structure as a binder-free anode for high-performance lithium-ion batteries. *Small*. **10**, 5035–5041 (2014).

10. Mukherjee, R., Thomas, A. V., Krishnamurthy, A. & Koratkar, N. Photothermally reduced graphene as high-power anodes for lithium-ion batteries. *ACS Nano*. **6**, 7867–7878 (2012).

11. Luo, J. *et al*. Three-dimensional graphene foam supported Fe_3O_4 lithium battery anodes with long cycle life and high rate capability. *Nano Lett*. **13**, 6136–6143 (2013).

12. Wei, W. *et al*. 3D graphene foams cross-linked with pre-encapsulated Fe_3O_4 nanospheres for enhanced lithium storage. *Adv. Mater.* **25**, 2909–2914 (2013).

13. Fang, Y. *et al*. Two-dimensional mesoporous carbon nanosheets and their derived graphene nanosheets: synthesis and efficient lithium ion storage. *J. Am. Chem. Soc*. **135**, 1524–1530 (2013).

14. Cao, X. *et al*. Metal oxide-coated three-dimensional graphene prepared by the use of metal-organic frameworks as precursors. *Angew. Chem. Int. Ed*. **53**, 1404–1409 (2014).

15. Gong, Y. *et al*. A bottom-up approach to build 3D architectures from nanosheets for superior lithium storage. *Adv. Funct. Mater*. **24**, 125–130 (2014).
16. Qin, J. *et al*. Graphene networks anchored with Sn@graphene as lithium ion battery anode. *ACS Nano*. **8**, 1728–1738 (2014).
17. Chen, R. *et al*. Free-standing hierarchically sandwich-type tungsten disulfide nanotubes/ graphene anode for lithium-ion batteries. *Nano Lett*. **14**, 5899–5904 (2014).
18. Xing, Z. *et al*. One-pot hydrothermal synthesis of Nitrogen-doped graphene as high-performance anode materials for lithium ion batteries. *Sci. Rep*. **6**, 26146 (2016).
19. Sun, H. *et al*. Three-dimensional holey-graphene/niobia composite architectures for ultrahigh-rate energy storage. *Science*. **356**, 599–604 (2017).
20. Lee, K. T., Lytle, J. C., Ergang, N. S., Oh, S. M. & Stein, A. Synthesis and rate performance of monolithic macroporous carbon electrodes for lithium-ion secondary batteries. *Adv. Funct. Mater.* **15,** 547–556 (2005).
21. Su, F. B., Zhao, X. S., Wang, Y., Wang, L. K. & Lee, J. Y. Hollow carbon spheres with a controllable shell structure. *J. Mater. Chem.* **16**, 4413–4419 (2006).
22. Hu, Y. S. *et al*. Synthesis of hierarchically porous carbon monoliths with highly ordered microstructure and their application in rechargeable lithium batteries with high-rate capability. *Adv. Funct. Mater*. **17**, 1873–1878 (2007).
23. Fang, B. Z. *et al*. Ordered multimodal porous carbon with hierarchical nanostructure for high Li storage capacity and good cycling performance. *J. Mater. Chem*. **20**, 10253–10259 (2010).
24. Chen, X. C. *et al*. Synthesis, growth mechanism, and electrochemical properties of hollow mesoporous carbon spheres with controlled diameter. *J. Phys. Chem. C*. **115**, 17717–17724 (2011).
25. Mao, Y. *et al*. Lithium storage in nitrogen-rich mesoporous carbon materials. *Energy Environ. Sci*. **5**, 7950–7955 (2012).
26. Li, D. D. *et al*. Novel nitrogen-rich porous carbon spheres as a high-performance anode material for lithium-ion batteries. *J. Mater. Chem. A*. **2**, 16617–16622 (2014).
27. Zhu, C. Y. & Akiyama, T. Cotton derived porous carbon via an MgO template method for high performance lithium ion battery anodes. *Green Chem*. **18**, 2106–2114 (2016).
28. Subramaniyam, C. M. *et al.* Self-assembled porous carbon microparticles derived from halloysite clay as a lithium battery anode. *J. Mater. Chem. A*., **5**, 7345–7354 (2017).
