# Supplementary material for: High-quality mesoporous graphene particles as high-energy and fast-charging anodes for lithium-ion batteries
Source: Nat Commun. 2019 Apr 1;10:1474. doi: 10.1038/s41467-019-09274-y (PMC6443805; doi:10.1038/s41467-019-09274-y)
Supplement: Supplementary file 3 — Description of Additional Supplementary Files [file 41467_2019_9274_MOESM3_ESM.pdf]

Description of Additional Supplementary Files

File Name: Supplementary Movie 1

Description: Lithiation and delithiation of HNMG to observe the structural stability and morphology evolution.
